# Reductive Methylation of Homogeneous Primary β-Lauryl/myristyl 7/3 Polyethyleneoxy n = 3–18 Ethylamines under Phase-Transfer Catalysis Conditions

**DOI:** 10.3390/molecules26154612

**Published:** 2021-07-29

**Authors:** Călin Jianu

**Affiliations:** Faculty of Food Engineering, Banat’s University of Agricultural Sciences and Veterinary Medicine “King Michael I of Romania” from Timisoara, Calea Aradului 119, 300645 Timișoara, Romania; calin.jianu@gmail.com

**Keywords:** reductive alkylation of amines, Leuckart–Wallach reaction, higher primary polyetheramines, aminative reduction, higher tertiary polyetheramines, homogeneous polyetheramines

## Abstract

Homogeneous tertiary *N*,*N*-dimethyl-*N*-β-lauryl/myristyl 7/3 polyethyleneoxy n = 3–18 ethylamines, LM(EO)_n_AT, are niche intermediates in the synthesis of homogeneous *N*-alkyl (C_1_–C_18_)-*N*,*N*-dimethyl-*N*-β-lauryl/myristyl 7/3 polyethyleneoxy n = 3–18 ethylammonium chlorides (unitary degree of oligomerization of ethylene oxide in the polyoxyethylene chain). This paper synthetically presents the dependence of the reductive methylation yields of homogeneous primary β-lauryl/myristyl 7/3 polyethyleneoxy n = 3–18 ethylamines, LM(EO)_n_AP, on the reaction time (10–90 min), the temperature (70 °C), the molar ratio formic aldehyde /LM(EO)_n_AP (1.1/1–2.5/1), the molar ratio HCOOH/LM(EO)_n_AP (5/1), the degree of oligomerization of ethylene oxide in the homogeneous polyoxyethylene chain in the 3,6,9,12,18 series, and the structure of the phase-transfer catalysts. The steric effects of hydrophobic groups CH_3_ and C_18_H_37_ grafted onto the ammonium function, and the micellar phenomena in the vicinity of their critical micellar concentration, directly proportional to the homogeneous degree of oligomerization, were highlighted. In all cases, a steady increase in reductive methylation yields was observed, with even quantitative values obtained. The high purity of the homologous series LM(EO)_n_AT will allow their personalization as reference structures for the study of the evolution of basic colloidal characteristics useful in forecasting technological applications. LM(EO)_n_AP were obtained either by direct amidoethylation (nucleophilic addition under basic catalysis of homogeneous lauryl/myristyl 7/3 polyethoxylated n = 3, 6, 9, 12, 18 alcohols, LM(EO)_n_OH, to acrylamide monomer) or by cyanoethylation of LM(EO)_n_OH under basic catalysis at 25–50 °C, in the presence of Fe^2+^ cations as oligomerization/polymerization inhibitor, followed by partial acid hydrolysis of homogeneous β-alkyl (C_12_H_25_/C_14_H_29_) 7/3 polyethyleneoxy n = 3, 6, 9, 12, 18 propionitriles, LM(EO)_n_PN, to β-alkyl (C_12_H_25_/C_14_H_29_) 7/3 polyethyleneoxy n = 3, 6, 9, 12, 18 propionamides, LM(EO)_n_PD, which led to LM(EO)_n_AP by Hoffmann degradation. Homogeneous higher tertiary polyetheramines LM(EO)_n_AT were structurally characterized.

## 1. Introduction

**Figure 1:**
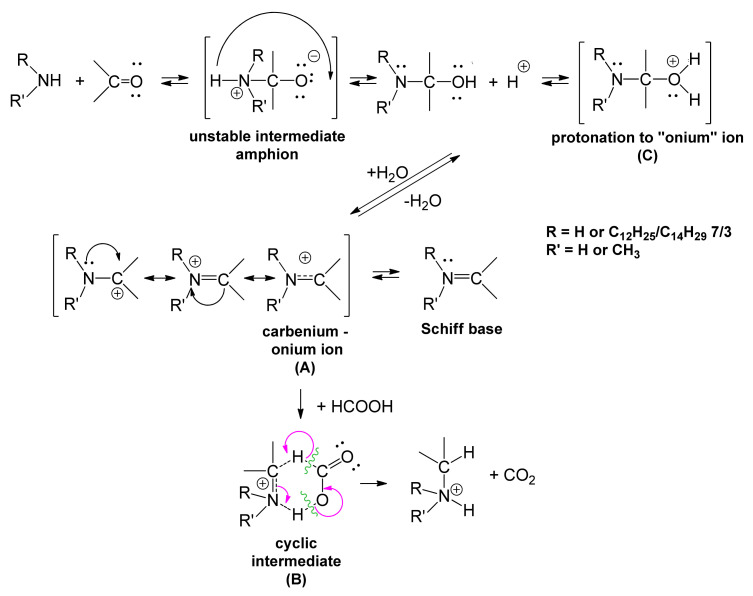
Schematic representation of the mechanism of reductive methylation of homogeneous primary β-lauryl/myristyl polyethyleneoxy n = 3–18 ethylamines, LM(EO)_n_AP [3].

Reductive amination, also known as “aminative reduction” or “reductive alkylation” for almost 150 years [1,2], is an oxidation-reduction process (mostly heterogeneous with a pronounced ionic character) for grafting alkyl (including methyl), alkylaryl, aryl, hetaryl, etc. radicals to the primary/secondary amino functional group. It is a process appreciated in organic synthesis for the formation of new C–N covalent bonds, based on the affinity of carbonyl compounds (aldehydes and ketones) towards nucleophilic addition and the reducing action of formic acid [3], formates [4], silanes [5], and borohydrides [6] such as: NaBH_3_CN—sodium cyanoborohydride; NaBH(OAc)_3_—sodium triacetoxyborohydride etc.; or formamide. The recent literature in the field mainly considers two ways of carrying out the process: (a) indirect reductive alkylation (amination) or (b) direct reductive alkylation (amination) [7]. If formic acid is used as a reducer, we can distinguish the Wallach reaction [1], and if we additivate the alkylation process with ammonium salts of formic acid, ammonium tetrafluoroborate (NH_4_BF_4_), ammonium perchlorate (NH_4_ClO_4_), ammonium hexafluorophosphate (NH_4_PF_6_), or formamide, we distinguish the Leuckart reaction [2]. Both processes in the original (classic) version have major disadvantages (average yields, mixtures of secondary and tertiary amines along with non-alkylated primary ones, formation of *N*-formyl derivatives above 180 °C), which limited the generalization of these processes. The reductive methylation that employed formic aldehyde and formic acid has become widespread, the Eschweiler–Clarke reaction. The steadily growing interest in reductive methylation studies has been driven, with all the limitations mentioned, by the fact that tertiary amino groups are generally frequently present in biologically active compounds (biologically active structures with added value) in modern medicine, pharmaceutical synthesis, compounds specialized in the elimination of CO_2_, of sulfur impurities, monitored polymerization, stabilization of polymers against degradation induced by light radiation, etc. [8,9,10]. The synthesis of tertiary amines is highly known according to the reviewed literature [11,12,13,14,15,16,17] through the following three operating protocols: (a) benzyne intermediates; (b) organometallic reagents; and (c) cross-coupling reactions. The following arguments are made against this popularity: the high number of benzyne precursors, the expensive access to coordinating metal catalysts (ruthenium, rhodium, platinum, palladium, nickel, cobalt, zinc, etc.), the toxicity of the solvents, etc. During the same period, different reaction mechanisms [3] were proposed for Leuckart–Wallach reactions, wherein imines, iminium ions, *N*-formyl imines, *N*-formyl iminium cations, carbenium-onium ions, and amphions appear (Figure 1). The reactivity of aldehydes and ketones is due to the advanced polarization of the electron cloud in the C=O double bonds by -I_s_; -E_s_ effects [11,12].

The literature [3] reports representative examples of reductive amination, of which we selectively mention benzaldehyde/methylamine (η = 90%); benzaldehyde/aniline (η = 90%); benzaldehyde/β-phenethylamine (η = 70%); furfural/ammonia (η = 50%); acetophenone/ammonia (η = 80%); benzyl methyl ketone/methylamine (η = 80%); cyclohexanone/ammonia (η = 80%); and cyclohexanone/cyclohexylamine (η = 70%). If the process is performed with formic aldehyde and formic acid as reducing agent, we find the specific parameters of the Leuckart–Wallach reaction, when the carbonyl compound (formic aldehyde) initially adds the nucleophilic amine, subsequently forming the corresponding Schiff base. The azomethine cation (carbenium-onium ion) (Figure 1A) is reduced with formic acid through a cyclic intermediate (Figure 1B) with the release of CO_2_ and the formation of the secondary amine, then, after resuming the cycle, of the tertiary amine [3].

**Figure 2:**
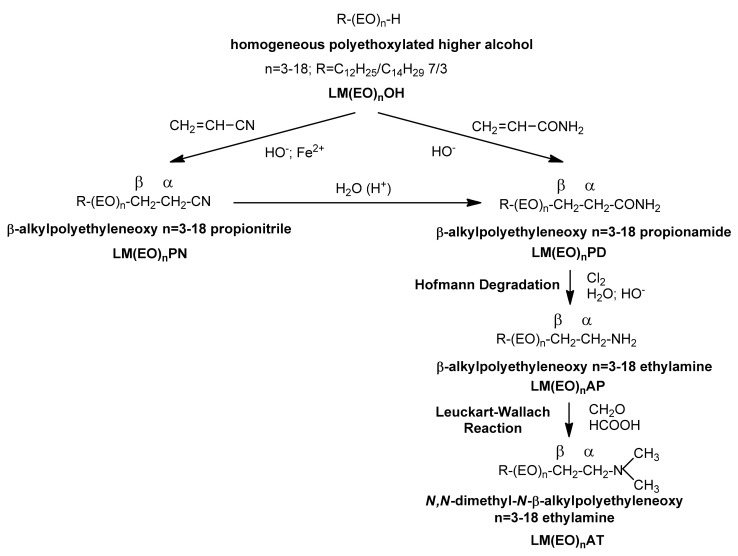
Reaction scheme for obtaining homogeneous tertiary *N*,*N*-dimethyl-*N*-β- lauryl/myristyl 7/3 polyethyleneoxy n = 3–18 ethylamines, LM(EO)_n_AT, by reductive methylation.

Conventional *N*-alkylation (including methylation) with alkyl halides, *O*-alkylation and/or *S*-alkylation have also benefited from the competencies of phase-transfer catalysis [13,14,15], allowing the unfolding of processes with increased yields in aqueous media, even without the exclusive participation of strong Lewis bases. Protonation of the amphionic intermediate (Figure 1C) takes place at the interface separating the organic phase from the aqueous one, but not exclusively. The most accessed phase-transfer catalysts cited in the literature have been *N*,*N*,*N*-tricapryl-*N*-methylammonium chloride, *N*,*N*,*N*,*N*-tetrabutylammonium chloride, and *N*,*N*,*N*-triethyl-*N*-benzylammonium chloride. Soluble in both phases, they transfer the low energy carbenium-onium cation with positive charge delocalized in both directions. Among the most publicized examples, we mention aliphatic amines as such, aromatic, or mixed: hydrazobenzene, phenylhydrazone, diazoamine derivatives, and nitrogen heterocycles (pyrrole, pyrrolidone, pyrazole, imidazole, benzimidazole, substituted aziridines, isoxazolones, pyridines, morpholine, indole, benzotriazole, carbazole, etc.) [3,16]. In essence, although steady progress has been made over the last century in understanding the specificity and mechanism of the reductive alkylation (methylation) process, it can be stated with certainty that the interest was focused on aliphatic, aromatic, and heterocyclic primary amines as such or mixed, lower or medium, while in the category of aldehydes, the lower/middle homologues of the series were studied primarily. The consulted literature does not report studies on higher primary/secondary amines or homogeneous higher primary/secondary polyether alkyl amines (with a strictly defined degree of oligomerization of ethylene oxide in the polyoxyethylene chain). In this sense, the major objective of this paper is to study the dependence of reductive methylation yields in the series of homogeneous polyoxyethylene chain homologues n = 3, 6, 9, 12, 18 (Figure 2) of homogeneous primary β-lauryl/myristyl 7/3 polyethyleneoxy n = 3–18 ethylamines, LM(EO)_n_AP, obtained by cyanoethylation or amidoethylation, i.e., nucleophilic addition under basic catalysis of homogeneous lauryl/myristyl 7/3 polyethyleneoxy n = 3, 6, 9, 12, 18 alcohols, LM(EO)_n_OH, [17,18] to acrylonitrile or acrylamide monomers, respectively (Figure 2) [19,20,21,22,23], followed by the partial hydrolysis of homogeneous β-lauryl/myristyl 7/3 polyethyleneoxy n = 3, 6, 9, 12, 18 propionitriles, LM(EO)_n_PN, or the Hofmann degradation of homogeneous β-lauryl/myristyl 7/3 polyethyleneoxy n = 3, 6, 9, 12, 18 propionamides, LM(EO)_n_PD, obtained, respectively, on the main operating parameters (temperature, molar ratio of reactants, reaction time, phase-transfer catalysis).

## 2. Results and Discussions

**Figure 3:**
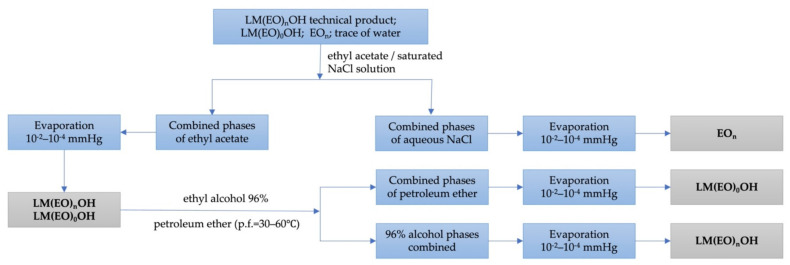
The block scheme of operations for the purification of LM(EO)_n_OH technical product from traces of water, LM(EO)_0_OH, and EO_n_ by repeated solid/liquid and liquid/liquid extractions.

The reductive methylation of LM(EO)_n_AP (not reported in the literature) is also an oxidation-reduction process with a pronounced heterogeneous ionic character, in which the direct contact of the reactants depends decisively on the homogeneity of the system. As a result, operating yields rarely exceed 80%. Preliminary experimental tests performed on the entire chain of transformations (Figure 2) with heterogeneous LM(EO)_n_OH, technical products, in an unprotected atmosphere (with O_2_, CO_2_, moisture) led even from the cyanoethylation stages to mixtures of complex compounds. This confirms the aspects reported previously and for the cyanoethylation of the same hydroxyl substrates without polyoxyethylene chains: the existence of an induction period (approx. 3–5 min), the subsequent appearance of yellow-orange suspensions, of reddish-brown resin, the violent and even explosive evolution of the process, the simultaneous unfolding of two parallel, concurrent reactions (nucleophilic addition/anionic polymerization of the acrylic monomer), and the reversible character at long processing times and/or high alkalinity. The subsequent strategy followed after cyanoethylation attempted to monitor the other steps (Figure 2) after the purification of LM(EO)_n_PN from secondary products generated by the mentioned aggression vectors by filtration, eluting on an open chromatographic column using solvent gradient systems, molecular distillation, etc., with inconclusive results. The exhaustive removal of O_2_, CO_2_, and moisture from the processing atmosphere was carried out with N_2_ atmosphere as inert gas (4–6 mL/min) transferred through batteries of processing columns filled with aqueous pyrogalol solution 40%, granular decarbonated KOH, and granular silica gel indicator impregnated with 2% cobalt chloride. Finely ground FeSO_4_ was suspended in the reaction mixture as polymerization inhibitor. The exhaustive preliminary purification of LM(EO)_n_OH from LM(EO)_0_OH, EO_n_, traces of water, secondary products consuming excess acrylic monomer, and subsequently other reagents was performed by repeated solid/liquid and liquid/liquid extractions in binary/ternary solvent systems (Figure 3).

**Figure 4:**
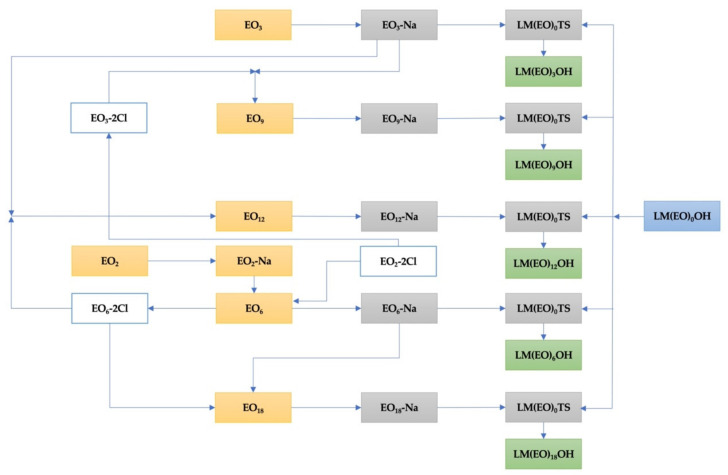
The step-by-step structuring scheme of homogeneous lauryl/myristyl 7/3 polyethyleneoxy n = 3, 6, 9, 12, 18 alcohols, where: EO_n_—homogeneous polyethylene glycols n = 3, 6, 9, 12, 18; EO_n_-Na—monosodium homogeneous polyethylene glycols; (EO)_n_-2Cl—dichlorinated homogeneous polyoxyethylene n = 3, 6, 9, 12 chains; LM(EO)_n_OH—homogeneous lauryl/myristyl 7/3 polyethyleneoxy n = 3, 6, 9, 12, 18 alcohols; LM(EO)_n_TS—tosylated homogeneous lauryl/myristyl 7/3 polyethoxylated n = 3, 6, 9, 12, 18 alcohols.

Subsequent experimental tests performed on the entire chain of transformations (Figure 2) with purified LM(EO)_n_OH (Figure 3) confirmed yields 10–15% higher than for LM(EO)_n_OH technical product, but also constantly increasing yields proportional to the size of the degree of oligomerization of ethylene oxide in the polyoxyethylene chain. The question was why, especially due to the degree of oligomerization, if the hypothesis of active participation of polyoxyethylene chains in the development of reactions is real, knowing the statistical distribution of chain homologues (Weibull/Nycander; Weibull/Nycander/Gold; Natta/Mantica; Poisson etc.) in LM(EO)_n_OH or the technical product. The origin of the constantly growing interest that has fascinated for more than a century the scientific efforts of researchers towards the special characteristics of polyoxyethylene chains in different situations was due to the specific structure and reactivity of the oxirane ring of ethylene oxide, the promoter of peaked ethoxylated alcohol [24] with a statistical distribution of polyether chain homologues, difficult to isolate by classical physico-chemical methods due to the neighboring effect and the sympathy effect. Laboriously, the homogeneous structure was made synthetically by the adapted Williamson method (Figure 4) of step-by-step attachment under phase-transfer catalysis, with higher yields, using high purity diethylene glycol and/or triethylene glycol as structural starting units.

From the simple working hypothesis (initially speculative) able to explain phenomena or processes, research based on X-ray investigations, microscopy, and electron diffraction has confirmed the ability of intra- and intercatenary contraction, dependent on structure, environment, with the formation of cavities (cages with variable, adaptable geometry), depending on the degree of oligomerization of ethylene oxide in polyoxyethylene chains with more than 8–9 oxyethylene units, and “sandwich” for chain sizes between 3–6 structural units, respectively [25,26,27,28,29].

The synergistic cumulation in a unitary structure of the conformational and colloidal competencies of these chains, with the possibility of directed modification of the hydrophilic/hydrophobic balance, remains in perspective of wide technological interest. Although pros and cons persist in the profile literature, experimental data associated with cross-interpretations provide sufficient constructive elements that support the free catenary rotation, the release of conformational tensions, and explain ways of packing (sequestration, coordination) in the macromolecular matrix of polyoxyethylene chains. For simplicity, the kinetic and energy efficiency of the phase-transfer catalysis was also becoming a major challenge for the study of the reductive methylation of homogeneous primary LM(EO)_n_AP.

**Figure 5:**
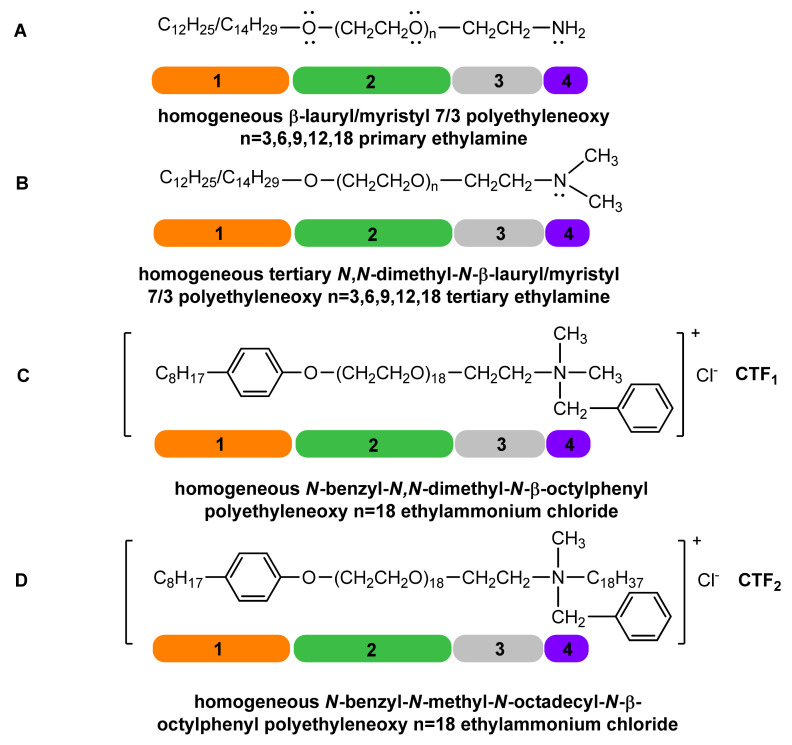
The functional structuring of homogeneous higher primary and tertiary alkyl polyetheramines (**A**,**B**) [30,31,32] and of certain phase-transfer catalysts (CTF_1_; CTF_2_) (**C**,**D**) [33]. 1—nonpolar hydrophobic group; 2—nonionic polar hydrophilic group; 3—connecting nonpolar hydrophobic group; 4—cationic polar hydrophilic group.

In the casuistics of reductive methylation of homogeneous primary LM(EO)_n_AP addressed in the study, preliminary tests confirmed the role of direct contact of the reactants (water solubility). This becomes a major working premise that also affects the subsequent processing strategies. The homologous series of homogeneous primary LM(EO)_n_AP occupies from this point of view the entire range of values of the hydrophilic/hydrophobic balance, W/O emulsions, wetting agents (wetting/spreading), O/W emulsions, washing agents, micellar stabilizers, as shown by their functional structural analysis (Figure 5A,B) [23,24,30,31,32].

The rediscovery of crown polyethers by Pedersen, A. [34] and of their sequestering (coordinating) characteristics was natural to generate the further question of which will be the behavioral (conformational) similarity with acyclic polyether chains (homogeneous and/or heterogeneous polyoxyethylene chains with varying degrees of oligomerization of ethylene oxide). Cumulatively interpreting the colloidal properties, the formation of polar micelles in polar media (water, formic acid) [35] with the conformational and ionic structural ones of the homogeneous polyoxyethylene chain and of the primary/tertiary amine, and quaternary ammonium functional groups of homogeneous LM(EO)_n_AP and LM(EO)_n_AT (Figure 5A,B), homogeneous *N*,*N*-dimethyl-*N*-benzyl-*N*-β-octylphenyl polyethyleneoxy n = 18 ethyl ammonium chloride [33] (Figure 5C) (CTF_1_), and *N*-methyl-*N*-benzyl-*N*-octadecyl-N-β-octylphenyl polyethyleneoxy n = 18 ethyl ammonium chloride [33] (Figure 5D) (CTF_2_), respectively, these operating premises can be argued in the proposed reductive methylation reaction. Preliminary experimental probes have confirmed the dependence of the processing yields of homogeneous LM(EO)_n_AP on the structure and nature of the reaction medium. Subsequently sequential evaluations also confirmed the following aspects.

An overview of the dependence of the reductive methylation yields of the homologous series of homogeneous LM(EO)_n_AP highlights constantly increasing yields compared to those reported for the structure of homogeneous LM(EO)_0_AP. The possible explanation may be the direct conformational participation of homogeneous polyoxyethylene chains in the processing, directly proportional to the size of the degree of oligomerization of ethylene oxide in the homogeneous polyoxyethylene chain. To confirm the hypothesis, the reductive methylation of homogeneous LM(EO)_n_AP was performedunder similar conditions (Table 1, Table 2 and Table 3). The obtained yield (79%) (statistical average) was below the value recorded for homogeneous LM(EO)_3_AP, the homologous series head (85%). The same experimental test was compared with the reductive methylation yield of homogeneous LM(EO)_18_AP, noticing an even more obvious difference (over 99%). These results support the hypothesis of conformational participation proportional to the magnitude of the degree of oligomerization of ethylene oxide in the nonionic structure. Following the same reasoning, the reductive methylation of the homogeneous LM(EO)_0_AP was performed under the same parameters, with the addition of 0.0001 moles phase-transfer catalyst (CTF_2_) (Figure 5D), relative to 0.1 moles homogeneous LM(EO)_0_AP. The yield 92% confirms the beneficial role of the phase-transfer catalyst through the polyoxyethylene chain n = 18, but also through the ammonium functional group. If the reductive methylation is performed under similar conditions with the addition of 0.0001 moles CTF_1_ (Figure 5C), the yield reaches 95%. The difference in yield between CTF_1_ and CTF_2_ is most likely due to the “steric hindrance” effect generated by the quaternary ammonium groups (the octadecyl radical in CTF_2_ versus the methyl one in CTF_1_). The participation of CTF_1_ and/or CTF_2_ in the reductive methylation reaction of homogeneous LM(EO)_0_AP can also be explained by the encapsulation of the onium intermediates (Figure 1), either in sandwich architectures for homogeneous polyoxyethylene chains n = 3 or in cage architectures (cavity, helix) for homogeneous polyoxyethylene chains n = 18. The same involvement is probably responsible for the sharp increase of the reductive methylation yields in the case of homogeneous LM(EO)_n_AP by the catalytic effect of internal phase transfer.

Phase-transfer catalysis achieves effective contact between the non-electronic substrate present in the organic phase, homogeneous LM(EO)_18_AP dissolved in the non-polar solvent suitable for the size of the homogeneous polyoxyethylene chain, and the ionic intermediates or polar reactants (formic aldehyde, formic acid) in the aqueous phase, separated by an interface in the system. In the studied casuistics, the transfer from the aqueous phase to the organic one was performed by CTF_1_ or CTF_2_, charged catalysts (quaternary ammonium salts structured in non-polar micelles), and simultaneously uncharged catalysts through the homogeneous polyoxyethylene chains n = 3–18 included in the same molecular assembly (Figure 5).

The process was successively accompanied by (a) interface phenomena dependent on the direct contact of the reactant structures (formic aldehyde, formic acid, and homogeneous primary polyetheramine) by effective mechanical stirring of the heterogeneous system. The mentioned processes became possible when the concentration of homogeneous LM(EO)_n_AP lay below the value of their critical micellar concentration, being quasimonomolecularly ordered at the interface separating the two phases of the heterogeneous system with the primary amino group in the aqueous (polar) phase, and by effectively stirring, the direct contact of the reactants was considerably favored by the increase of the system interface; (b) micellar phenomena catalyzed by the quaternary ammonium functional group in the CTF_1_ or CTF_2_ surface-active structures in micelles with hydrophobic (non-polar) cavities in the aqueous phase and hydrophilic (polar) cavities in the organic phase, respectively. The interphase displacement of CTF_1_ or CTF_2_ was also accompanied by micellar inversion. In the development of micellar processes in the organic phase, both areas (with and without charge) in the CTF_1_ structure participated simultaneously through specific competencies, providing the direct contact between homogeneous primary polyetheramine/formic aldehyde and formic acid to initiate the mechanism of reductive methylation (Figure 1). The kinetics of the process were considerably accelerated in the vicinity and above the critical micellar concentration (Table 4 and Table 5).

## 3. Materials and Methods

### 3.1. Chemicals

Diethylene glycol (EO_2_), triethylene glycol (EO_3_), acrylonitrile, acrylamide, acetic anhydride, *p*-toluene sulfochloride (TsCl), thionyl chloride (SO_2_Cl_2_), and chromatographic purity solvents were purchased from Sigma-Aldrich Chemie GmbH (Hamburg, Germany). LM(EO)_0_OH was purchased from Condea Chemie GmbH (Hamburg, Germany) (Table 6). All substances were used as received.

### 3.2. Synthesis and Characterization of Reference Chemicals

Homogeneous LM(EO)_n_OH (Table 6); homogeneous LM(EO)_n_AP (Table 7) (Figure 2); homogeneous LM(EO)_n_PN (Figure 2); homogeneous LM(EO)_n_PD (Figure 2); homogeneous polyethylene glycols n = 3, 6, 9, 12, 18, EO_n_, (Table 8); mono- and diacetylated polyethylene glycols n = 3, 6, 9, 12, 18, (EO)_n_-2Ac, (Table 9); mono- and disodium homogeneous polyethylene glycols (EO)_n_-Na and (EO)_n_-2Na (Table 10); tosylated homogeneous lauryl/myristyl 7/3 polyethoxylated n = 3, 6, 9, 12, 18 alcohols, LM(EO)_n_TS; homogeneous tertiary *N*,*N*-dimethyl-*N*-β-lauryl)/myristyl 7/3 polyethyleneoxy n = 3–18 ethylamines (Table 11) (Figure 5B); and homogeneous dichlorinated polyethylene glycols n = 3–18 (Table 12) were synthesized according to previously published procedures with minor modifications [19,20,21,22,23]. The phase-transfer catalysts, homogeneous *N*,*N*-dimethyl-*N*-benzyl-*N*-β-octylphenyl polyethyleneoxy (n = 18) ethylammonium chloride (CTF_1_) (Figure 5C), and homogeneous *N*-methyl-*N*-benzyl-*N*-octadecyl-*N*-β-octylphenyl polyethyleneoxy n = 18 ethylammonium chloride (CTF_2_) (Figure 5D), were synthesized according to previously published procedures with modifications [33]: catalytic hydrogenation of LM(EO)_n_PN at 100–150 °C, 10–15 atm, catalyst PdO_3_ deposited on activated carbon, and polar protic solvent.

### 3.3. Chemical and Physico-Chemical Characterization of Homogeneous Lauryl/myristyl Alcohols 7/3 as such and Polyethoxylated n = 3–18

The chemical and physico-chemical characterization was performed by using previously reported experimental conditions [40,41] (Table 6).

### 3.4. The Overall Scheme of Synthesis Reactions of Homogeneous LM(EO)_n_AT from Homogeneous LM(EO)_n_OH

For the synthesis of homogeneous LM(EO)_n_AT, the reaction scheme in Figure 2 was applied using previously reported experimental conditions [19,20,21,22,23,24].

### 3.5. Synthesis and Characterization of Homogeneous LM(EO)_n_AT under Conditions of Phase-Transfer Catalysis

In a reaction vessel with a lid provided with effective mechanical stirring, a controlled dosing system of the reactants, and an ascending refrigerant mounted in the refrigeration bath of a cryostat, were introduced successively a 20–30% solution of 0.1 moles homogeneous LM(EO)_n_AP, in the non-polar organic solvent selected depending on the degree of oligomerization of ethylene oxide in the homogeneous polyoxyethylene chain (n = 3–18), then under continuous stirring at 5–15 °C (controlled temperature) 0.5 moles formic acid 85% aqueous solution, followed by the solution of 0.12 moles formic aldehyde relative to each methyl group subsequently grafted on the homogeneous LM(EO)_n_AP and 0.0001 moles homogeneous *N*,*N*-dimethyl-*N*-benzyl-*N*-β-octylphenyl polyethyleneoxy (n = 18) ethylammonium chloride (CTF_1_) or homogeneous *N*-methyl-*N*-benzyl-*N*-octadecyl-*N*-β-octylphenyl polyethyleneoxy (n = 18) ethylammonium chloride (CTF_2_), respectively. The system was heated approx. 2–4 h (compared to 10–12 h in the classic procedure). As a guide, the reaction was considered complete after the cessation of CO_2_ emissions. The processing mixture was acidified with 35% HCl in the presence of Congo red indicator after which it was evaporated to dryness under vacuum (10^−2^–10^−4^ mm Hg). The obtained residue was dissolved/suspended in a minimum amount of cold water. Free homogeneous tertiary *N*,*N*-dimethyl-*N*-β-lauryl/myristyl 7/3 polyethyleneoxy n = 3–18 ethylamines were subsequently isolated by controlled addition of aqueous NaOH (KOH) 25% and purified by repeated extractions (2–3 times) with anhydrous ethyl ether. The ether phases, combined and thoroughly dehydrated by transfer to columns filled with anhydrous Na_2_SO_4_, were evaporated. Free homogeneous LM(EO)_n_AT were purified subsequently by molecular distillation (10^−4^–10^−7^ mm col Hg) and structurally characterized (Table 11).

## 4. Conclusions

The reductive methylation of homogeneous LM(EO)_n_AP under conditions of phase-transfer catalysis can be considered an achieved goal, a sustainable synthesis variant, accessible for derivatization to homogeneous LM(EO)_n_AT. The high yields and their purity do not suggest limits and restrictions and offer the real possibility of further diversification for new nonionic-ionic (cationic) surface-active structures, broadening the niche spectrum of these hybrid molecular architectures. The encouraging results also allow the process to be generalized to other higher primary amines, with the contribution of the phase-transfer catalysis conferred by homogeneous *N*-methyl-*N*-benzyl-*N*-alkyl(C_1_-C_18_)-*N*-β-octylphenyl polyethyleneoxy n = 3–18 ethylammonium chlorides CTF_2_ and others.

## Figures and Tables

**Table 1:** The dependence of the reductive methylation yield of LM(EO)_n_AP on the degree of oligomerization of ethylene oxide in the polyoxyethylene chain n = 3, 6, 9, 12, 18, reaction time 90 min, molar ratio formic aldehyde/LM(EO)_n_AP 1.3/1, molar ratio formic acid/LM(EO)_n_AP 5/1, and temperature 70 °C.

No.	Degree of Oligomerization (n)	Reductive Methylation Yield (%)
1	3	81.32
2	6	86.42
3	9	90.17
4	12	92.36
5	18	95.02

**Table 2:** The dependence of the reductive methylation yield of LM(EO)_3;18_AP on the reaction time, molar ratio formic aldehyde/LM(EO)_3;18_AP 1.3/1, molar ratio formic acid/LM(EO)_3;18_AP 5/1, and temperature 70 °C.

No.	Degree of Oligomerization (n)	Reaction Time (Minute)	Reductive Methylation Yield (%)
1	3	10	31.22
2		30	47.15
3		50	62.51
4		90	80.83
5	18	10	49.02
6		30	60.61
7		50	77.43
8		90	94.61

**Table 3:** The dependence of the reductive methylation yield of LM(EO)_3;18_AP on the molar ratio formic aldehyde/LM(EO)_3;18_AP, reaction time 50 min, molar ratio formic acid/LM(EO)_3;18_AP 5/1, and temperature 70 °C.

No.	Degree of Oligomerization (n)	Molar Ratio Formic Aldehyde /LM(EO)_3;18_AP	Reductive Methylation Yield (%)
1	3	1.1/1	59.43
2		1.3/1	62.54
3		1.7/1	69.61
4		2.0/1	76.32
5		2.2/1	90.52
6		2.5/1	99.14
7	18	1.1/1	74.32
8		1.3/1	77.13
9		1.7/1	92.20
10		2.0/1	94.03
11		2.2/1	97.74
12		2.5/1	99.25

**Table 4:** Critical micellar concentration of the phase-transfer catalyst homogeneous *N*,*N*-dimethyl-*N*-benzyl-*N*-β-octylphenyl polyethyleneoxy n = 18 ethylammonium chloride (CTF_1_) (Figure 5C).

No.	Homogeneous Degree of Oligomerization, n, of Ethylene Oxide in the Structure a CTF_1_	Critical Micellar Concentration (×10^–5^ mol/L)
1	3	1.31
2	6	1.11
3	9	0.82
4	12	0.53
5	18	0.40

**Table 5:** Critical micellar concentration of homogeneous tertiary *N*,*N*-dimethyl-*N*-β-lauryl/myristyl 7/3 polyethyleneoxy n = 3–18 ethylamines, LM(EO)_n_AT (Figure 2 and Figure 5B).

No.	Homogeneous Degree of Oligomerization, n, of Ethylene Oxide in the Structure LM(EO)_n_AT	Critical Micellar Concentration (×10^–5^ mol/L)
1	3	1.43
2	6	1.22
3	9	0.94
4	12	0.64
5	18	0.49

**Table 6:** The main physico-chemical characteristics of homogeneous lauryl/myristyl 7/3 alcohols as such and homogeneous lauryl/myristyl 7/3 polyethoxylated n = 3, 6, 9, 12, 18 alcohols, LM(EO)_n_OH, purity 98%.

Alcohol	Physico-Chemical Characteristics				
	Density (g/cm^3^)/Temperature (°C)	Solidification Range, (°C)	Clouding Point [36,37], (°C)	Hydroxyl Value [38,39], (mg KOH/g)	Ethylene Oxide Content [40,41], (%)
Lauryl/myristyl alcohol	0.830/25	33–35	-	-	-
Polyethoxylated lauryl/myristyl alcohol (n = 3)	0.891/70	-	-	180.41	42.52
Polyethoxylated lauryl/myristyl alcohol (n = 6)	0.933/70	20–23	46–50	126.58	59.67
Polyethoxylated lauryl/myristyl alcohol (n = 9)	0.943/70	26–28	58–67	97.49	68.94
Polyethoxylated lauryl/myristyl alcohol (n = 12)	0.966/70	31–34	87–96	79.27	74.74
Polyethoxylated lauryl/myristyl alcohol (n = 16)	0.975/70	36–38	>100	63.45	79.78
Polyethoxylated lauryl/myristyl alcohol (n = 18)	0.984/70	38–40	>100	52.91	83.14

**Table 7:** The main chemical characteristics of homogeneous primary β-lauryl/myristyl 7/3 polyethyleneoxy n = 3–18 ethylamines, LM(EO)_n_AP.

No.	Primary Polyether Amine	Nitrogen Content [42,43], (%)		Ethylene Oxide Content [40,41], (%)	
		Experimental	Theoretical	Experimental	Theoretical
1	β-lauryl/myristyl 7/3 oxy-ethylamine	5.884	3.897	-	-
2	β-lauryl/myristyl 7/3 polyethyleneoxy n = 3 ethylamine	3.770	3.790	35.545	35.733
3	β-lauryl/myristyl 7/3 polyethyleneoxy n = 6 ethylamine	2.781	2.792	52.449	52.652
4	β- lauryl/myristyl 7/3 polyethyleneoxy n = 9 ethylamine	2.201	2.210	62.268	62.520
5	β-lauryl/myristyl 7/3 polyethyleneoxy n = 12 ethylamine	1.818	1.829	68.594	68.983
6	β lauryl/myristyl 7/3 polyethyleneoxy n = 16 ethylamine	1.482	1.487	74.546	74.782
7	β-lauryl/myristyl 7/3 polyethyleneoxy n = 18 ethylamine	1.250	1.253	78.582	78.757

**Table 8:** The main chemical and physico-chemical characteristics of homogeneous polyethylene glycols, (EO)_n_, n = 3, 6, 9, 12, 18.

No.	Symbol	Ethylene Oxide Content [40,41], (%)		Hydroxyl Value [38,39], (mg KOH/g)		Refractive Index, $n_{D}^{40}$	
		Determined	Calculated	Determined	Calculated	Determined	Calculated
1	PEG—3	87.204	88.000	370.294	373.330	-	-
2	PEG—6	92.480	93.617	106.168	198.501	1.4523	1.4520
3	PEG—9	94.772	95.650	130.024	135.256	1.4593	1.4591
4	PEG—12	95.886	96.703	101.698	102.564	1.4606	1.4608
5	PEG—18	97.080	97.770	68.639	69.135	1.4626	1.4628

**Table 9:** The main chemical characteristics of several homogeneous polyoxyethylene chains (EO)_n_ n = 3, 6, 9, 18 mono- (EO)_n_—Ac) and diprotected (EO_n_—2Ac) by acetylation.

No.	Homogeneous Degree of Oligomerization (n)	EO_n_—Ac						EO_n_—2Ac					
		Ethylene Oxide Content [40,41], (%)		Hydroxyl Value [38,39], (mg KOH/g)		Purity (%)		Ethylene Oxide Content [40,41], (%)		Hydroxyl Value [38,39], (mg KOH/g)		Purity (%)	
		Detd.	Calcd.	Detd.	Calcd.	Detd.	Calcd.	Detd.	Calcd.	Detd.	Calcd.	Detd.	Calcd.
1	3	68.56	68.75	290.88	291.67	99.73	55.78	56.41	1.782	-	98.90	3	68.56
2	6	81.11	81.48	172.05	172.84	99.54	70.32	71.93	1.803	-	97.76	6	81.11
3	9	86.21	86.84	121.93	122.81	99.28	79.17	79.52	1.647	-	99.56	9	86.21
4	18	89.05	89.79	94.45	95.24	99.17	83.54	83.81	1.025	-	99.68	12	89.05

**Table 10:** The main chemical characteristics of several homogeneous polyoxyethylene chains (EO)_n_ n = 3, 6, 9, 18, (EO)_n_-Na) and (EO)_n_-2Na.

No.	Homogeneous Degree of Oligomerization (n)	(EO)_n_—Na		(EO)_n_—2Na	
		Purity (%)		Purity (%)	
		Determined ^1^	Calculated	Determined ^1^	Calculated
1	3	99.04	99.18	99.18	99.79
2	6	99.37	99.86	99.03	99.77
3	9	99.43	99.88	98.99	99.83
4	18	99.78	99.53	99.36	99.88

^1^ reporting the experimental values to the calculated ones (theoretical); alkalinity assessment (mol/L 10^−3^) by acid-base titration in anhydrous, inert medium.

**Table 11:** The main chemical characteristics of homogeneous tertiary *N*,*N*-dimethyl-*N*-β-lauryl/myristyl 7/3 polyethyleneoxy n = 0, 3, 6, 9, 12,18 ethylamines, LM(EO)_n_AT.

No.	Homogeneous Tertiary Polyether Amine	Ethylene Oxide Content [40,41], (%)		Nitrogen Content [42,43], (%)	
		Experimental	Theoretical	Experimental	Theoretical
1	*N*,*N*-dimethyl-*N*-β-lauryl/myristyl 7/3 oxy ethylamine	-	-	5.247	5.275
2	*N*,*N*-dimethyl-*N*-β-lauryl/myristyl 7/3 polyethyleneoxy n = 3 ethylamine	33.061	33.216	3.505	3.522
3	*N*,*N*-dimethyl-*N*-β-lauryl/myristyl 7/3 polyethyleneoxy n = 6 ethylamine	46.619	49.867	2.631	2.644
4	*N*,*N*-dimethyl-*N*-β-lauryl/myristyl 7/3 polyethyleneoxy n = 9 ethylamine	59.704	59.873	2.111	2.117
5	*N*,*N*-dimethyl-*N*-β-lauryl/myristyl 7/3 polyethyleneoxy n = 12 ethylamine	66.294	66.549	1.758	1.765
6	*N*,*N*-dimethyl-*N*-β-lauryl/myristyl 7/3 polyethyleneoxy n = 18 ethylamine	76.270	76.829	1.213	1.222

**Table 12:** The main chemical characteristics of dichlorinated homogeneous polyoxyethylene (n = 3, 6, 9, 12) chains (EO)_n_- 2Cl.

No.	Homogeneous Degree of Oligomerization (n)	(EO)_n-_2Cl			
		Ethylene Oxide Content [40,41], (%)		Chlorine Content [44,45], (%)	
		Determined	Calculated	Determinated	Calculated
1	3	56.67	57.14	30.48	30.74
2	6	72.42	72.73	19.48	19.56
3	9	79.59	80.00	14.27	14.34
4	12	84.06	84.21	11.30	11.32

## Data Availability

The data presented in this study are available on request from the corresponding author.

## Sample Availability

Samples of the compounds are available from the author.
